# Comparative In Vitro Study of the Effect of Er:YAG Laser on the Debonding of Lithium Disilicate Veneers With Two Different Surface Treatments

**DOI:** 10.1155/ijod/9946208

**Published:** 2026-04-08

**Authors:** Ayham Halloum, Rima Saker, Omar Hamadah

**Affiliations:** ^1^ Department of Fixed Prosthodontics, Faculty of Dentistry, Tishreen University, Latakia, Syria, tishreen.edu.sy; ^2^ Department of Oral Medicine, Faculty of Dentistry, Damascus University, Damascus, Syria, damascusuniversity.edu.sy

**Keywords:** ceramic veneers, debonding, Er:YAG laser, lithium disilicate

## Abstract

**Aim:**

To conduct a comparative study of lithium disilicate veneers treated with two different methods, hydrofluoric acid and Er:YAG laser, and to evaluate the role of the Er:YAG laser in their debonding time. Additionally, to examine bonding failure modes of veneers after debonding by both methods.

**Methods and Materials:**

The research sample consisted of 20 permanent human maxillary central incisors; they were divided randomly into two equal groups and prepared to receive lithium disilicate veneers. The surfaces of the veneers in the first group were treated with 9.5% hydrofluoric acid for 90 s, while those in the second group were treated with the Er:YAG laser with a wavelength of 2940 nm, a frequency of 15 Hz, an energy of 400 mJ, and a water/air cooling ratio of 8:4 for 20 s. Subsequently, veneers in both groups were bonded with resin cement. The Er:YAG laser (with a wavelength of 2940 nm, a frequency of 10 Hz, an energy of 400 mJ, and a water/air cooling ratio of 1:1) was used in a non‐contact mode to debond the veneers, recording the time from the start of laser application until debonding. The bonding failure modes for all samples were then evaluated using a stereomicroscope under a 20× magnification. Data were analyzed with SPSS, a *t*‐test was used to compare debonding time, and a chi‐square test was applied to analyze failure modes.

**Results:**

Statistically significant differences were found in the debonding time of veneers using the Er:YAG laser between the two groups (*p* < 0.05). The hydrofluoric acid group showed a shorter mean debonding time 114.2 ± 37.22 s compared to the Er:YAG laser group 187.3 ± 64.49 s. Additionally, no statistically significant differences were observed in the frequencies of bonding failure modes of veneers between the two groups (*p* > 0.05) with adhesive failure at the veneer–cement interface being the most common type.

**Conclusions:**

Lithium disilicate veneers treated with an Er:YAG laser require a longer time to debond compared to those treated with hydrofluoric acid when using the non‐contact mode of the Er:YAG laser. The bonding failure mode was similar for both methods, showing adhesive failure between the veneer’s internal surface and the resin cement.

## 1. Introduction

Ceramic veneers have gained wide popularity for improving the aesthetics of anterior teeth, as they are conservative, long‐lasting restorations with high aesthetic quality [1]. However, complications such as margin fractures, discoloration, improper placement of the restoration, patient dissatisfaction, or other reasons may necessitate the removal of these restorations [2]. Successful removal depends on maintaining the integrity of the ceramic veneer and also keeping the dental tissues undamaged beyond repair [3, 4]. Removing failed ceramic veneers typically involves the use of rotary instruments, which makes the procedure lengthy and often results in the loss of some intact tissue, especially because of the minimal color difference among tooth, cement, and veneer layers [5, 6].

A new technique has been developed as an alternative to the traditional method using rotary instruments for debonding ceramic veneers, employing Erbium lasers. This method offers the advantage of potentially reusing the removed veneers if they remain intact, thereby reducing time and cost [7]. Studies have shown that laser beams can pass through the ceramic and can be selectively absorbed by water molecules and residual monomers in the luting cement, leading to the evaporation of water particles, subsequent expansion and increase in volume beneath the ceramic surface due to vapor formation, and the occurrence of micro‐explosions, resulting in a hydrodynamic ejection that helps in debonding the veneer [8]. The type and thickness of the ceramic, the type of resin cement used, the laser parameters applied, and the surface treatment methods all influence the removal process [7, 9]. The traditional method for treating the inner surface of glass ceramics involves hydrofluoric acid treatment [10], which dissolves the glassy matrix and produces micro‐irregularities on the ceramic surface, forming small pits that enhance surface roughness and improve the effectiveness of subsequent silane application [11, 12]. The use of hydrofluoric acid to etch glass ceramics can compromise their mechanical integrity. Variations in both the duration of etching and the concentration of the acid determine the extent and morphology of surface irregularities, which in turn influence the bonding performance and strength of the ceramic. When these irregularities are excessive or uneven, particularly if the cement does not fully cover the intaglio surface, the likelihood of ceramic fracture increases [13]. Additionally, hydrofluoric acid poses a potential health hazard to dentists due to its toxicity; it can cause harm to the skin or eyes if accidentally contacted or irritate the dentist’s lungs over the long term due to its volatility [14, 15].

Recently, various types of lasers, including Nd:YAG, Er, Cr:YSGG, Er:YAG, and CO_2_ lasers, have been studied for conditioning the inner surfaces of different types of ceramics to improve bonding with resin cement [16–21]. Previous studies have indicated that when the surface of the ceramic crown is exposed to Erbium laser irradiation, structural removal occurs through a thermomechanical mechanism. The water contained within the ceramic or supplied by the cooling system absorbs the laser energy, undergoes rapid vaporization, and generates localized micro‐explosions that consequently alter the surface structure [21–23].

The effect of laser irradiation on ceramic surfaces depends largely on the chemical composition and the microstructural characteristics of the irradiated ceramic. Through precise control of laser parameters, selective surface modification can be achieved without adversely affecting the internal structure or mechanical properties of the ceramic. However, the use of high energy levels or inappropriate settings may result in irregular surface alterations or the formulation of microcracks, which may compromise the structural integrity of the ceramic [22, 24].

Given the limited literature on this subject, the objective of this in vitro study is to compare the use of the Er:YAG laser and evaluate its role and extent of effect in the debonding of lithium disilicate veneers treated with two different methods (hydrofluoric acid and Er:YAG laser) and to compare the bonding failure modes with both methods.

## 2. Methods and Materials

The Protocol of the study had been approved by the Ethics Committee of the Collage of Dentistry Research Centre at Tishreen University under approval [3945] during session [22], held on June 20, 2023.

Due to the lack of previous studies directly comparing Er:YAG laser debonding of lithium disilicate veneers following two different surface treatments, an a priori sample size calculation was not feasible. Therefore, a sample size of 20 specimens (10 per group) was used.

### 2.1. Inclusion Criteria

Permanent human maxillary central incisors similar in size.

### 2.2. Exclusion Criteria

Teeth presenting dental caries, cracks, fractures, or previous restorations as well as teeth not stored in physiological saline were excluded.

**Figure 2:**
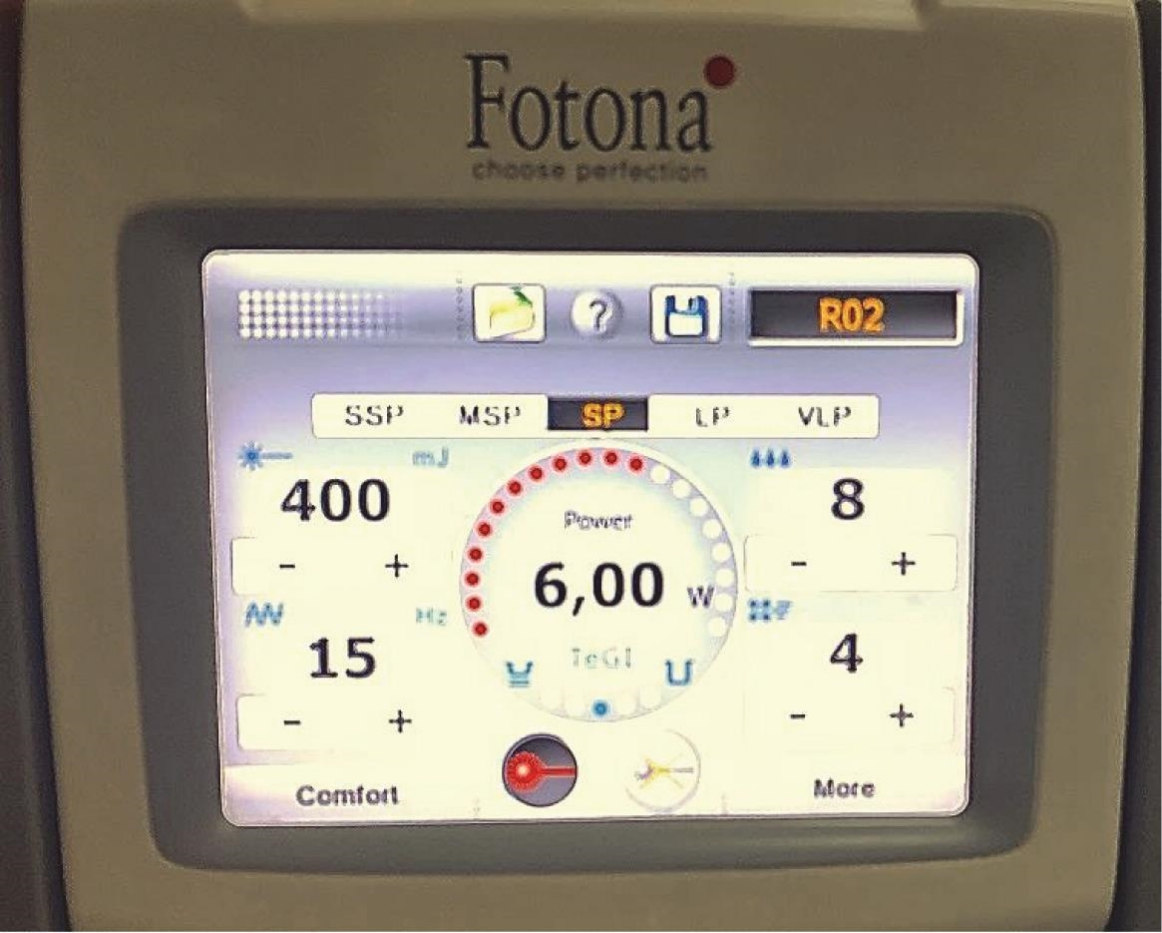
Screen of the Er:YAG laser device with the parameters used for treating the surface of the lithium disilicate veneer.

• Twenty permanent human maxillary central incisors extracted due to periodontal disease were collected, cleaned, and stored in physiological saline at room temperature with daily replacement of the solution until used.
 They were placed in acrylic molds, leaving the crown and 2 mm of the root exposed, with the labial surface perpendicular to the horizontal plane.
• Randomization was performed using the online software www.graphpad.com/quickcalcs/randomize1/. All experimental procedures, including tooth preparation, surface treatment, bonding, and debonding, were performed by a single calibrated operator to minimize inter‐operator variability.
• All teeth were prepared with a unified preparation area for receiving the lithium disilicate veneers, based on a specific outline on parchment paper with a gingival‐incisal length of 10 mm, a mesiodistal width of 9 mm in the incisal third, 8 mm in the middle third, and 7 mm in the cervical third, to be applied to all sample teeth to standardize the prepared surface area.
• The preparation thickness was standardized for all teeth using a depth‐cutting bur, with the labial surfaces prepared to a depth of 0.5 mm in the cervical third and 0.7 mm in the middle and incisal thirds, and a 0.5 mm chamfer finish line at the cementoenamel junction. The incisal edge was prepared using a butt joint design (Figure 1).
• All preparations were performed by a single operator following the same standardized protocol.
• Since the teeth were placed in acrylic molds, custom impression trays were fabricated, all with identical dimensions.
• Impressions were taken using the double impression technique in one stage using heavy‐ and light‐bodied addition silicone (Variotime, Kulzer, Germany) and sent to the laboratory for the fabrication of veneers from lithium disilicate IPS e.Max Press (Ivoclar, Vivadent; Schaan, Liechtenstein) in A3 shade according to the manufacturer’s instructions and with standardized thickness and area for all veneers.

• The central incisors were randomly divided into two equal groups:
   Group 1: (Hydrofluoric acid treatment group): The veneers in this group were treated with 9.5% hydrofluoric acid (Porcelain Etchant, Bisco, USA) for 90 s (according to the manufacturer’s instructions and based on the findings of Porto et al. [25], who reported that this etching duration produced higher surface roughness and more stable shear bond strength on lithium disilicate ceramic), followed by rinsing with water and drying with air.
   Group 2: (Er:YAG laser treatment group): The veneers in this group were treated with the Er:YAG laser (Fotona‐Lightwalker; Slovenia, 2011) with a wavelength of 2940 nm, a non‐contact mode, a frequency of 15 Hz, an energy of 400 mJ, and a water/air cooling ratio of 8:4, and in short pulse mode (SP), using a scanning motion at a distance of ~7 mm for 20 s (Figure 2). These parameters were selected for their effectiveness in producing adequate surface roughness on lithium disilicate ceramic following the findings of Yepo et al. [26].

The bonding steps were subsequently completed identically for both groups as follows:

Figure 1(A) Image showing the research sample of 20 prepared maxillary incisors for receiving lithium disilicate veneers. (B) Close‐up image of the preparation of one of the sample teeth to highlight the details of the preparation.

**Figure:**
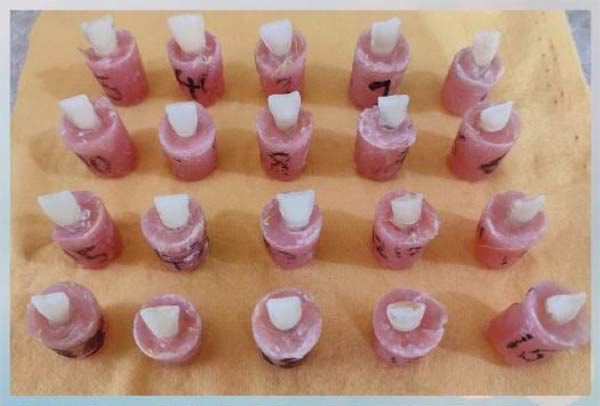
(A)

**Figure:**
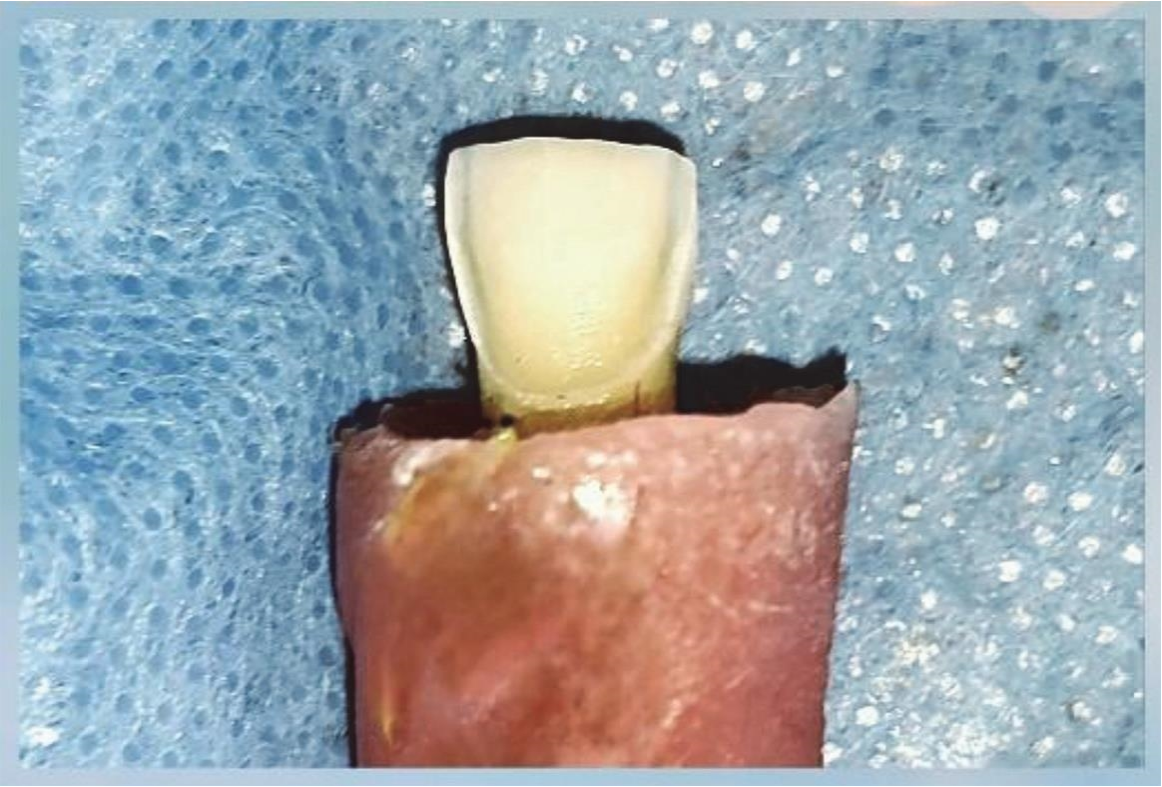
(B)

**Figure 3:**
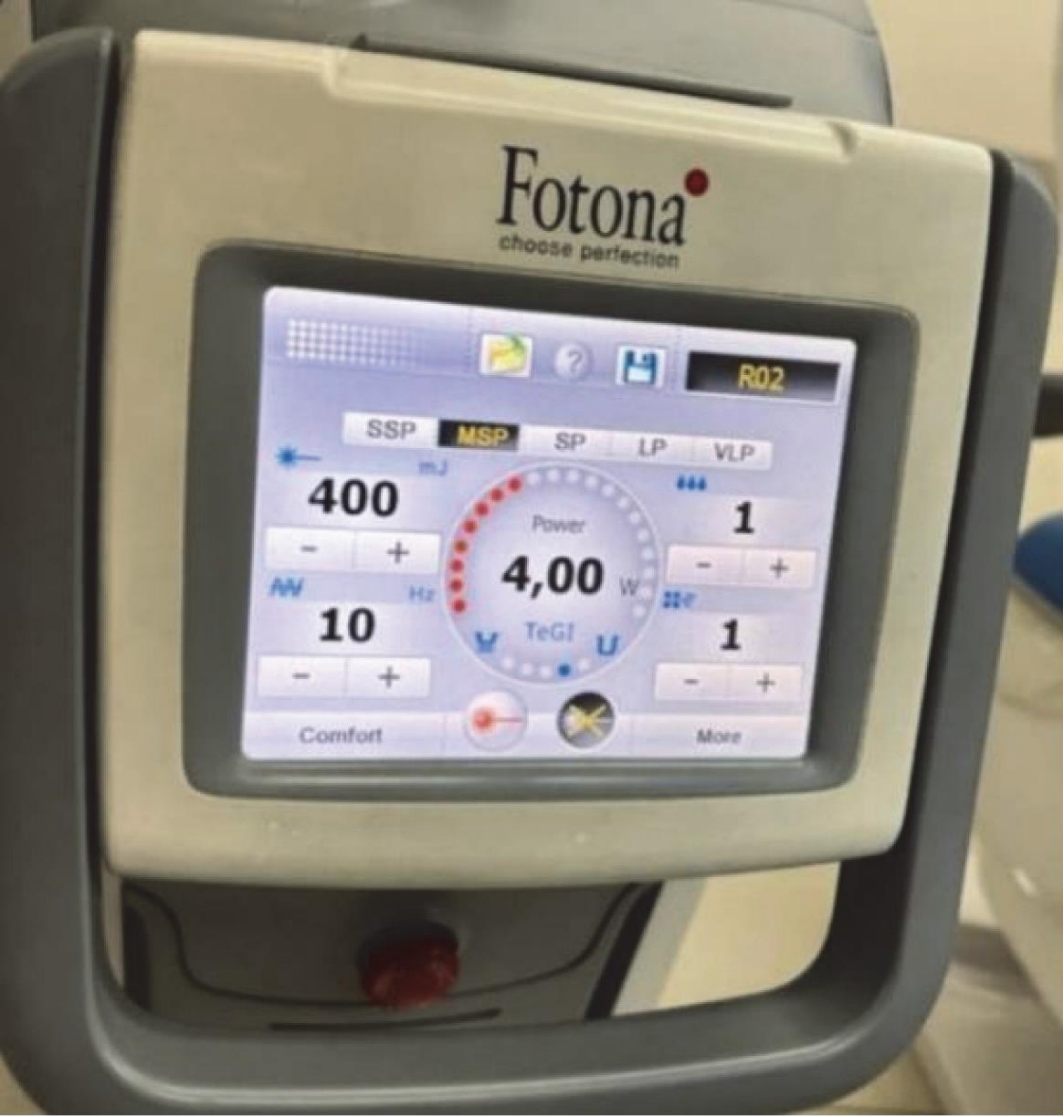
Screen of the Er:YAG laser device with the parameters used for debonding the veneers.

• Two layers of porcelain primer (PORCELAIN PRIMER, BISCO, USA) were applied to the inner surface of the veneer according to the manufacturer’s instructions, left for 30 s, then air‐dried.
• 37% phosphoric acid (ETCH‐37, BISCO, USA) was applied according to the manufacturer’s instructions, followed by rinsing and drying.
• Two layers of the bonding agent (ALL‐BOND UNIVERSAL, BISCO, USA) were applied to the etched tooth surface with a brush for 10 s for each layer without light‐curing between the layers, followed by air‐blowing for 10 s and then light‐curing according to the manufacturer’s instructions.
• Light‐cured resin cement (CHOICE 2 Light Cured Veneer Cement, BISCO, USA) was applied in a uniform amount to the inner surface of the veneer with a milky bright shade to all veneers, then placed on the prepared tooth and pressed with a mirror holder in the middle of the cosmetic veneer. Light curing was performed for 3 s, excess cement was removed, and curing was continued for 40 s on all sides.

• All cemented samples were stored in distilled water at a 37° C for 48 h before using the Er:YAG laser to debond veneers.
• The Er:YAG laser (Fotona‐Lightwalker; Slovenia, 2011) was used to debond the veneers with a wavelength of 2940 nm in non‐contact mode with a frequency of 10 Hz, an energy of 400 mJ, and a water/air cooling ratio of 1:1, and in medium‐short pulse mode (MSP) (Figure 3), at a distance of ~7 mm from the surface of the lithium disilicate veneer and with a scanning method in a horizontal direction from the mesio‐cervical margin to the disto‐occlusal margin. Afterwards, the scanning method application was repeated vertically until debonding occurred. At the same moment, a stopwatch was started by an assisting dentist, and the time in seconds from the moment of laser application until veneer debonding occurred was recorded in special tables.
• In the present study, intrapulpal temperature changes during laser debonding were not directly measured. The Er:YAG laser parameters applied were selected based on a previously study that demonstrated that similar settings did not result in harmful temperature increases.
• After debonding the ceramic veneers, both the inner surface of the veneer and the bonded tooth surface were examined under a 20× magnification using a stereo microscope (Euromex, Netherlands) (Figure 4) to investigate the failure modes, categorized into three types according to the modified criteria described by Al‐Balkhi et al., based on the classification proposed by Mak (2002):
  • Type 1: Adhesive failure between the inner surface of the lithium disilicate veneer and the resin cement, when most of the cement remains on the tooth surface.
  • Type 2: Adhesive failure between the tooth surface and the resin cement, when most of the cement remains on the inner surface of the lithium disilicate veneer.
  • Type 3: Cohesive failure within the resin cement.

All data related to failure modes were also recorded in special tables.

Figure 4(A) Examination of the tooth surface under 20× magnification. (B) Examination of the inner surface of the lithium disilicate veneer under 20× magnification.

**Figure:**
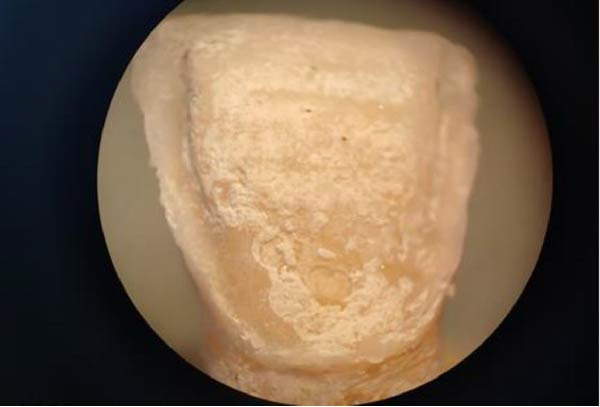
(A)

**Figure:**
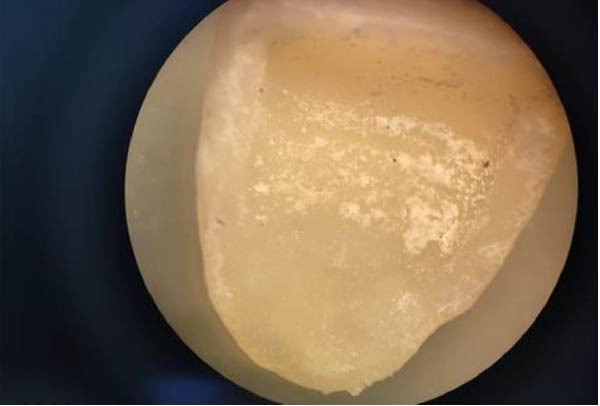
(B)

After confirmation of data normality using the Kolmogorov–Smirnov test, parametric analysis (independent *t*‐test) was used to compare debonding time between the two groups. Failure modes were analyzed using the non‐parametric chi‐square test. With a significance level set at *α* = 0.05.

## 3. Results

### 3.1. Study of the Debonding of Lithium Disilicate Veneers Using the Er:YAG Laser After Surface Treatment With Two Different Methods (Treatment With Hydrofluoric Acid and Treatment With the Er:YAG Laser)

The descriptive statistics and *p*‐Value for the debonding time are presented in Table 1. The results showed a statistically significant difference in the mean debonding time between the two surface treatment methods (*p* < 0.05). The Er:YAG laser‐treated group exhibited a longer debonding time compared to the hydrofluoric acid‐treated group.

**Table 1:** The descriptive statistics and *p*‐Value for the debonding time (in seconds) of lithium disilicate veneers according to the surface treatment method.

Surface treatment method	Variable studied = Debonding time of lithium disilicate veneers (in seconds) using Er:YAG laser					*p*‐Value
	Mean	Standard deviation	Standard error	Minimum	Maximum	
Er:YAG laser	187.30	64.49	20.39	120	280	^∗^0.006
Hydrofluoric acid	114.20	37.22	11.77	60	172	

^∗^Statistically significant differences.

#### 3.1.1. Effect Size and Confidence Intervals

The effect size for the difference in debonding time between the two surface treatment methods was 1.39, indicating a large effect according to Cohen’s conventions. Additionally, the mean difference in debonding time was 73.10 s with a 95% confidence interval of [23.6, 122.6] s.

### 3.2. Study of the Failure Mode of the Lithium Disilicate Veneers After Debonding

Failure mode analysis revealed that debonding predominantly occurred as type 1 adhesive failure in both groups, as shown in Table 2. There were no statistically significant differences in the frequency of failure modes for lithium disilicate veneers removed using the Er:YAG laser and those removed using hydrofluoric acid.

**Table 2:** Failure mode of veneers after debonding: number and percentage [*n* (%)] and *p*‐Value.

Surface treatment method	Number of veneers + (Percentage)			*p*‐Value
	Adhesive failure (ceramic‐resin)	Adhesive failure (Tooth‐resin)	Cohesive resin failure	
Er:YAG laser	9 (90%)	0	1 (10%)	^∗^0.531
Hydrofluoric acid	8 (80%)	0	2 (20%)	

^∗^No statistically significant differences.

## 4. Discussion

The present in vitro study compared the debonding time and failure modes of lithium disilicate veneers subjected to two different surface treatments: hydrofluoric acid and Er:YAG laser. The results demonstrated that veneers treated with an Er:YAG laser required significantly longer debonding time compared to those treated with hydrofluoric acid; however, no significant differences were observed in the frequencies of bonding failure modes of veneers between the two groups.

In the current study, maxillary central incisors were prepared to receive the lithium disilicate veneers because they are one of the most indicated teeth for veneers and can achieve the highest aesthetic outcomes [27]. The butt joint preparation design was selected among other incisal edge preparations because it showed higher failure rates and allowed for multiple insertion paths, which can lead to improper seating during the bonding process, possibly necessitating the removal and repositioning of the veneers after bonding [28, 29]. Lithium disilicate ceramic was chosen for its popularity in enhancing color matching and achieving high aesthetics, as well as its favorable mechanical properties [30, 31]. The Er:YAG laser was selected for debonding the veneers instead of the Er, Cr:YSGG laser because the Er:YAG laser is absorbed by water three times more effectively than the Er, Cr:YSGG laser, as shown in studies. This higher absorption results in shallower penetration depth, requiring less energy and time for tissue removal [32]. ALBalkhi et al. [9] study indicated that an energy level of 400 mJ and a frequency of 10 Hz were the most appropriate among various parameters for debonding lithium disilicate veneers treated with hydrofluoric acid, leading to the selection of these parameters for the current study on both surface treatment methods. Non‐contact mode was used instead of contact mode to reduce the time required [9].

In recent years, as the use of lasers for debonding veneers has become more common, studies have investigated the effectiveness of lasers in debonding under various conditions, including different laser parameters [9, 33–36], types and thicknesses of ceramic [4, 5, 37–39], and types of resin cements [40, 41]. However, the only traditional method for treating lithium disilicate ceramic surfaces has been hydrofluoric acid treatment. With the advancement of ceramic surface treatment techniques and the emergence of lasers as a modern method in this field to ensure bond strength, this study was conducted. The laser was used with an energy of 400 mJ, a frequency of 15 Hz, and a water/air cooling ratio of 8:4 due to the effectiveness of these parameters in creating good surface roughness for lithium disilicate ceramic surfaces, as shown by the study of Yepo et al. [26].

A resin cement in the milky bright shade was used for bonding the veneers, which were chosen in a dark shade (A3) to easily distinguish the remaining cement on the tooth surface and the inner surface of the veneer after debonding, facilitating the identification and classification of failure modes under a stereo microscope.

The results of the current study showed statistically significant differences in the mean debonding time of veneers between the two groups studied (treatment with hydrofluoric acid and treatment with Er:YAG laser). The mean debonding time was 114.2 and 187.3 s, respectively. The longer debonding time for the group treated with Er:YAG laser could be attributed to the micro‐roughness created on the surface of lithium disilicate ceramic when treated with Er:YAG laser. This roughness may increase the bonding surface area and thus enhance the ability of the cement to penetrate and wet the treated surface more effectively than the surface treated with hydrofluoric acid.

Although hydrofluoric acid remains the conventional surface treatment for lithium disilicate ceramics, data regarding laser‐conditioned ceramic surfaces and their behavior during laser‐assisted debonding are rare. To the best of the authors’ knowledge, the present study is the first to comparatively evaluate the Er:YAG laser debonding of lithium disilicate veneers treated with Er:YAG laser versus conventional hydrofluoric acid surface treatment.

The results of the current study for the mean debonding time of veneers treated with hydrofluoric acid were consistent with the in vitro study by Elenany et al. [42], which was conducted to debond ceramic veneers made of lithium disilicate e.Max CAD using an Er:YAG laser with two different powers (3 W and 5.4 W). The mean debonding time was 108.23 and 110.23 s, respectively. The current study’s results also aligned with Morford et al. study [43], where the mean debonding time for IPS e.Max veneers was 100 s. However, our results differed from Al‐Balkhi et al. study [9], where the mean debonding time was 15.38 s when using the laser with an energy of 400 mJ and a frequency of 10 Hz. This very short time compared to the current study can be attributed to Al‐Balkhi’s use of 15 N weight attached to a specially designed cervical prominence on the facial surface to apply tensile force on the ceramic veneer during laser application, which was not used in the current study to simulate clinical reality. Additionally, our results differed from Zhang et al. study [44], where the mean debonding time was 328 s, possibly due to the different parameters used in their study.

Several studies have been conducted to investigate the effect of surface treatment methods on the bond strength between resin cement and lithium disilicate ceramics. The current study aligns with the findings of Yepo et al. [26], which demonstrated that lithium disilicate ceramics treated with the Er:YAG laser exhibited higher bond strength compared to those treated with hydrofluoric acid. Meanwhile, Ergun‐Kunt et al. [45] evaluated the effects of various surface treatments on bond strength, including hydrofluoric acid treatment and 5 W Er:YAG laser application. They concluded that the use of the Er:YAG laser provided bond strength similar to that achieved with hydrofluoric acid treatment.

Although temperature changes were not directly measured in the present study, the laser parameters were selected based on the study of Al‐Balkhi et al. [9], who demonstrated that these parameters did not result in thermal damage to the tooth structure. In addition, as emphasized by Zhang et al. [44] when the laser is pulsed, the actual irradiation time is considerably shorter than the total working time. The long relaxation time intervals between pulses allow effective heat dissipation, thereby minimizing accumulation. Nevertheless, the lack of direct temperature measurement represents a limitation of this study and should be considered when interpreting its clinical applicability, and future studies are recommended to assess temperature changes during debonding.

The study of bond failure when using the laser for debonding is a significant detail, as it indicates the depth of laser penetration within the layers of the ceramic veneer, resin cement, and tooth surface, which provides an important indicator of potential enamel damage. The current study showed no statistically significant differences in the failure mode between the group treated with the Er:YAG laser and the group treated with hydrofluoric acid. Most samples in both groups exhibited Type 1 failure, which is adhesive failure between the inner surface of the lithium disilicate veneer and the resin cement. This could be attributed to the greater impact of the laser radiation at the bonding area between the resin cement and the inner surface of the lithium disilicate veneer, where the failure mode was also observed. This finding is consistent with most previous studies showing Type 1 failure [6, 7, 33, 42].

All veneers removed in this study remained intact after debonding, as confirmed by stereomicroscopic evaluation. Although this assessment method is limited, similar overall findings have been reported in literature using more advanced evaluation techniques. Zhang et al. [44] demonstrated that debonding by Er:YAG laser did not adversely affect the mechanical properties of lithium disilicate ceramics when appropriate parameters were used, and Grzech‐Leśniak et al. [46] observed no microstructural damage under scanning electron microscopy following repeated Er:YAG laser debonding and rebonding cycles. Collectively, these findings suggest that using an Er:YAG laser for veneer debonding may preserve the structural integrity of lithium disilicate veneers. Nevertheless, the absence of scanning electron microscopic evaluation in the present study represents a limitation, as stereomicroscopic assessment may not detect microcracks induced by laser irradiation. Therefore, future studies should be conducted to examine the structure of the debonded lithium disilicate veneers using a scanning electron microscope to explore the possibility of reusing them.

Limitations of this in vitro study include the relatively limited sample size, the absence of direct intrapulpal temperature measurement, and the lack of scanning electron microscopic evaluation.

## 5. Conclusions

Within the limits of this study, we find that:

• Lithium disilicate veneers treated with the Er:YAG laser require a longer time to debond compared to those treated with hydrofluoric acid when using the non‐contact mode of the Er:YAG laser.
• When using the Er:YAG laser to debond lithium disilicate veneers, adhesive failure between the inner surface of the veneer and the resin cement (Type 1 failure) occurred in both surface treatment methods.

## Author Contributions

**Ayham Halloum**: conceptualization, methodology, investigation (tooth preparation, surface treatment, bonding, and debonding procedures), data curation, formal analysis, writing – original draft. **Rima Saker**: conceptualization, study design, interpretation of results, writing – review and editing. **Omar Hamadah**: supervision, writing – review and editing, critical revision. All the authors read and approved the final version of the manuscript.

## Funding

The authors received no specific funding for this work.

## Ethics Statement

The Protocol of the study had been approved by the Ethics Committee of the Collage of Dentistry Research Centre at Tishreen University under approval [3945] during session (22), held on June 20, 2023.

## Consent

A written informed consent was obtained from all patients who underwent tooth extraction, allowing the use of their teeth in this study.

## Conflicts of Interest

The authors declare no conflicts of interest.

## Data Availability

The data that support the findings of this study are available from the corresponding author upon reasonable request.
